# Rapid whole genome sequencing of critically ill pediatric patients from genetically underrepresented populations

**DOI:** 10.1186/s13073-022-01061-7

**Published:** 2022-05-24

**Authors:** Nour Halabi, Sathishkumar Ramaswamy, Maha El Naofal, Alan Taylor, Sawsan Yaslam, Ruchi Jain, Roudha Alfalasi, Shruti Shenbagam, Martin Bitzan, Lemis Yavuz, Hamda Abulhoul, Shiva Shankar, Dalwinder Janjua, Devendrasing Jadhav, Munira Mahmoud Al Maazmi, Walid Abuhammour, Alawi Alsheikh-Ali, Mohamed Al Awadhi, Abdulla Al Khayat, Ahmad N. Abou Tayoun

**Affiliations:** 1 Al Jalila Genomics Center of Excellence, Al Jalila Children’s Specialty Hospital, Dubai, United Arab Emirates; 2 Kidney Center of Excellence, Al Jalila Children’s Specialty Hospital, Dubai, United Arab Emirates; 3 General Pediatrics Department, Al Jalila Children’s Specialty Hospital, Dubai, United Arab Emirates; 4 Department of Metabolic Medicine, Al Jalila Children’s Specialty Hospital, Dubai, United Arab Emirates; 5 Critical Care Centre of Excellence, Al Jalila Children’s Specialty Hospital, Dubai, United Arab Emirates; 6 Infectious Diseases Department, Al Jalila Children’s Specialty Hospital, Dubai, United Arab Emirates; 7 College of Medicine, Mohammed Bin Rashid University of Medicine and Health Sciences, Dubai, United Arab Emirates; 8 Dubai Health Authority, Dubai, United Arab Emirates; 9 Al Jalila Children’s Specialty Hospital, Dubai, United Arab Emirates; 10 Center for Genomic Discovery, Mohammed Bin Rashid University of Medicine and Health Sciences, Dubai, United Arab Emirates

## Abstract

We describe a case series of five infants (age range: 1–90 days; 4 females and 1 male) who presented to Al Jalila Children’s intensive care units (ICU) with complex multisystem disorders. Patients were Emirati, Kenyan, Jordanian, Filipino, or Pakistani. Trio rapid whole genome sequencing (rWGS) was performed on all five patients and their parents within the hospital’s genomics facility. Results were returned within ~37 h from blood sample draws and were diagnostic in 3 out of 5 patients. Positive findings were a homozygous pathogenic variant in *POMT1* gene causing muscular dystrophydystroglycanopathy, a mosaic tetrasomy of the short arm of chromosome 12 (12p13.33p11.1) causing Pallister-Killian syndrome, and compound heterozygous pathogenic variants in the *LIPA* gene causing lysosomal acid lipase deficiency and Wolman disease. The rWGS analysis provided fast and precise diagnostic findings in those 3 patients and also aided in devising better management plans for them in the intensive care setting. For example, the 3-month-old infant with pathogenic variants in the *LIPA* gene is now a candidate for an FDA-approved, potentially lifesaving enzyme replacement therapy (sebelipase alfa). Our case series emphasize the feasibility and utility of rWGS in pediatric intensive care setting, in a diverse population that has long been underserved in genomic services. Significant investments in local healthcare infrastructure are needed, globally, for more equitable access of genomic medicine among vulnerable patients.

**Supplementary Information:**

The online version contains supplementary material available at 10.1186/s13073-022-01061-7.

Rapid whole genome sequencing (rWGS) has recently been shown to provide timely diagnoses and management plans for critically ill patients in intensive care settings [1, 2]. Yet, access to such service requires significant investments in genomic infrastructure within local healthcare institutions, thereby limiting its worldwide application to patients who critically need it. Consequently, the global distribution of rWGS is highly unequal with implementation in a number of centers within Europe, the USA, and Australia [2], and lack of such service in other geographical regions such as the Middle East and Africa where the genetic disease burden, specifically recessive disorders, is expectedly high due to inbreeding [3, 4]. This also limits our understanding of the utility of rWGS for critically ill patients in those regions which tend to be significantly underrepresented in global genetic databases. For example, data from the Middle East account for only 0.01% of genome-wide association studies (GWAS) and less than 1% of all publicly accessible sequencing datasets [3, 4].

We therefore established a workflow for rWGS (Fig. 1) in the intensive care unit (ICU) at Al Jalila Children’s Specialty Hospital, a tertiary pediatric center in the United Arab Emirates caring for a diverse patient population of Middle Eastern, North African, and Asian origins which is historically not well represented in genetic studies [3, 4]. Our workflow involved a multidisciplinary team of pediatricians, neonatologists, and genetic counselors for case selection, patient consenting, return of results, and patient management. Selected candidates were either unstable with complex medical findings necessitating rapid diagnosis to aid in treatment or were admitted for an extended period without improvement despite previous comprehensive testing which remained inconclusive (Additional file 1: Methods).

**Fig. 1:**
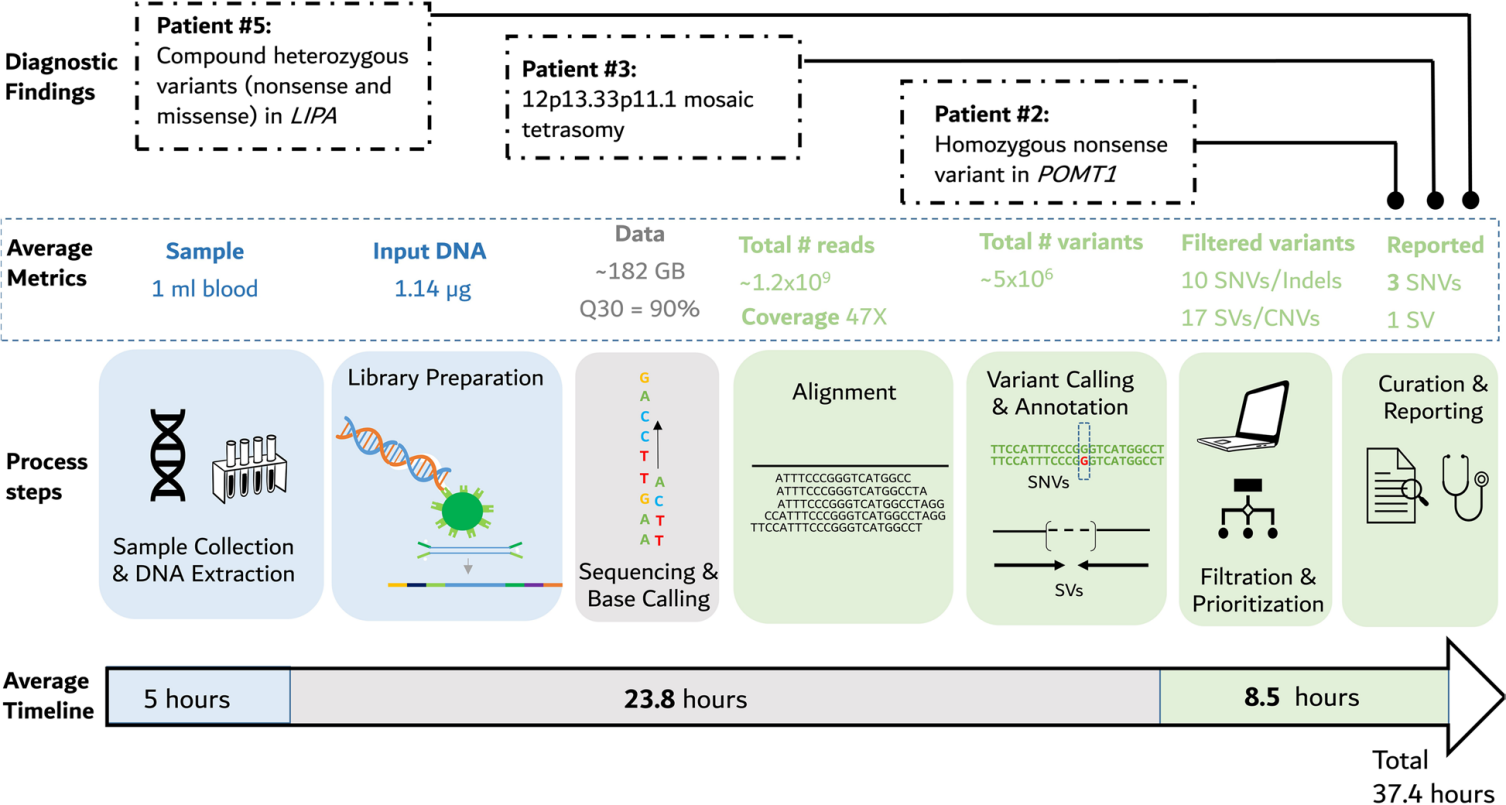
Overall step-by-step workflow of the rapid whole sequencing (rWGS) protocol along with a timeline and data metrics/requirements at each step. Average data from 5 patients/families are displayed. Detailed data are available in the supplemental files, tables, and figures. Molecular findings in patients #2, #3, and #5 are also shown. SNVs, single nucleotide variants; INDELs, small insertions and deletions; SVs, structural variants including copy number variants and short tandem repeats

Blood samples obtained from consented families were referred to the CAP-accredited genomics facility within the hospital which included a team of molecular technologists, genomic analysts, bioinformatic scientists, genetic counselors, and board-certified clinical molecular geneticist. After DNA extraction and library preparation, sequencing was performed using the Illumina NovaSeq 6000 System. Bioinformatic analysis, variant annotation, and filtration were performed using the Illumina TruSight Software Suite (TSS) [5] (Additional file 1: Methods). Variants were clinically interpreted following the American College of Medical Genetic and Genomics (ACMGG) guidelines [6]. Candidate variants were immediately communicated with the care team to reach a consensus diagnosis. A genetic counselor reviewed the results with all families and, in the case of a positive result, a multidisciplinary team met with the family to review the results and possible treatment plans. For positive cases, an additional consent was also obtained for clinical confirmatory testing whose results were shared with the medical team and directly with the family.

Five patients (4 females; average age, 28 days [range, 1–90 days]), who were either Emirati, Filipino, Jordanian, Kenyan, or Pakistani (Additional file 1: Table S1), presenting to the neonatal or pediatric ICU between September 2021 and February 2022, were enrolled for rWGS on research basis. All families provided informed consent after a counseling session with a board-certified genetic counselor. Parental samples were included for trio analysis. An initial diagnosis was obtained in 3 of the 5 patients with an average turnaround time of 37.3 h [range 35.8 to 39.4 h] (Fig. 1 and Additional file 1: Table S2).

Short read paired-end sequencing (2 X 150 base pairs) yielded, on average, 182 GB of data for ~1.2×10^9^ reads and an average autosomal coverage of 47X per genome (*n* = 15 genomes) (Additional file 1: Table S3). Alignment to the human reference genome (GRCh37) (average 94.5% of total reads) generated an average of 5,119,631 single nucleotide variants (SNVs), small insertions and deletions (indels), and structural variants (SVs) including copy number variants (CNVs) and short tandem repeats (STRs) (Additional file 1: Fig. S1). In addition, runs of homozygosity (ROH) across the autosome were calculated in each patient and showed that patients #2 and #4 had significant ROH (6.56% and 12%, respectively) which was consistent with reported consanguinity in both families (Additional file 1: Table S4).

Variant filtration and prioritization based on predicted protein effect, allele frequency, inheritance model, patient phenotype, and other features (Additional file 1: Methods and Fig. S1 and S2), reduced the number of candidate variants to 10 SNVs/Indels and 17 SVs, on average (Table S4). Overall, 3 SNVs and 1 structural variant met criteria for pathogenicity leading to a molecular diagnosis in 3 of the 5 patients (Fig. 1 and Additional file 1: Table S5). Each molecular diagnosis was immediately communicated to the treating physicians and the reported variants were found to be the primary cause for the 3 patients. All initial rWGS findings were subsequently clinically confirmed in the CAP-accredited laboratory within the hospital.

Patient #2, a 2-day-old premature Jordanian baby with antenatal findings including severe bilateral ventriculomegaly and cerebral hypoplasia, was transferred to the NICU from an outside hospital for surgical correction of imperforate anus at day 2 of life. Other manifestations included respiratory distress, microphthalmia, macrocephaly, holoprosencephaly, and anorectal malformation. Whole genome sequencing was completed within 35 h and 47 min after enrollment (Additional file 1: Table S2) and revealed a diagnostic homozygous nonsense variant in *POMT1* gene [7] and a clinical diagnosis of muscular dystrophy-dystroglycanopathy. This finding was confirmed by clinical exome sequencing (Additional file 1: Table S5). Although treatment was not affected and care remained palliative, this finding avoided additional diagnostic workup, and the family was counseled regarding the prognosis and recurrence of this condition. Pre-implantation genetic testing (PGD) and prenatal testing options were reviewed with the family for future pregnancies. Parents were reportedly second cousins, and whole genome sequencing revealed ROH regions in ~6.56% of the autosome confirming this relationship (Additional file 1: Table S4).

Patient #3 is a 1-day-old Pakistani female who was born at 37 weeks with antenatal findings including hypoplastic right ventricle secondary to tricuspid atresia. Delivery was complicated due to fetal bradycardia, and the newborn was admitted to NICU with complex congenital cyanotic heart defect (hypoplastic right heart syndrome), acute respiratory failure, and imperforate anus. rWGS, performed in 36 h and 8 min (Additional file 1: Table S2), identified a mosaic tetrasomy of the short arm of chromosome 12 which was confirmed by clinical SNP chromosomal microarray testing (Additional file 1: Fig. S3 and Table S5). This finding confirmed a clinical diagnosis of Pallister-Killian syndrome [8] and guided management of the patient. The identification of this tetrasomy using rWGS demonstrates the additional value of this testing where exome sequencing would, most likely, not have detected this arrangement leading to significant delays in diagnosis until separately ordered clinical chromosomal microarray testing results are obtained.

Finally, patient #5 was a 3-month-old Filipino female infant who was previously healthy. She initially presented to the emergency department with fever, jaundice, blood in stool, and recurrent vomiting after feeding. Upon evaluation, she was subsequently admitted to the PICU with hemophagocytic lymphohistiocytosis (HLH), hepatosplenomegaly, and liver dysfunction. rWGS was performed within 36 h and 30 min (Additional file 1: Table S2) and revealed compound heterozygous variants in *LIPA* [9–11], which were subsequently clinically confirmed by Sanger sequencing. This finding suggested a clinical diagnosis of Wolman disease secondary to lysosomal acid lipase deficiency (Additional file 1: Table S5) and was confirmed by liquid chromatography mass spectrometry which showed pathologically reduced acidic lipase enzymatic activity in patient’s blood. This finding had a significant impact on this patient’s clinical management. Not only did it rule out a primary HLH disease which would have been treated differently, but this finding also presented this patient as a candidate for hematopoietic stem cell transplantation (HSCT) or an FDA-approved, potentially life-saving enzyme replacement therapy (sebelipase alfa) which is currently being pursued. Recurrence risks and testing options for future pregnancies were reviewed with the family.

Targeted single-gene testing, chromosomal microarrays, and/or whole exome sequencing is the current standard of care in the ICU setting in limited centers within the UAE and most of the Middle East. However, very few of those centers have local access to this testing, so it is often sent outside the country, leading to extended turnaround times of at least 3–4 weeks, delayed diagnoses, and, most importantly, inefficient communication of results with non-genetics providers who lack access to genetic counseling support.

Here, we present the first rWGS diagnostic case series from the UAE and, to our knowledge, from the Middle East. This study demonstrates the feasibility and clinical utility of performing rWGS locally for critically ill children from this genetically underrepresented population and highlights the need for investments in pediatric genomics within local healthcare institutions, in the Middle East [3, 4] and globally, to deliver timely diagnoses and management. Such investments should be allocated towards establishing specialized pediatric tertiary care centers and genomic facilities equipped with state-of-the-art technologies and computational infrastructure, along with building human capacity, mainly genetic counselors, molecular technologists, genomic analysts, bioinformatic scientists, clinical molecular geneticists, and specialized multidisciplinary pediatric teams to treat and manage those patients often presenting with rare complex disorders. Recruiting, training, and retaining such skillsets remains a major challenge in the Middle East [3, 4] and in other low resource regions. It is therefore expected that the rWGS service will be limited to highly specialized tertiary centers within each country, minimizing the costly investments in genomic facilities, which can exceed $2–3 million, and maximizing the benefits to patients with the ability to recruit and retain multidisciplinary skillsets within such centers.

Given the large amount of WGS data, several assumptions were made, and manual steps were removed to expedite the identification and timely delivery of clinically significant variants in this setting. For example, heterozygous variants in genes associated with a dominant mode of inheritance were removed although such variants can be associated with milder phenotypes or incomplete penetrance leading to possibly missed diagnoses. Copy number variants were also limited to those > 10kb. Furthermore, in initially prioritizing candidate variants, we rely on P or LP assertions in ClinVar which has reduced representation of such variation in non-European patients thus reducing the sensitivity of this approach. A middle ground approach between the expedited analysis in this and other rWGS studies [1, 2] would be to perform a tier 1 expedited analysis, followed by a tier 2 complete analysis which can take longer but can include more in-depth analysis and interpretation to avoid missing diagnoses.

Despite those limitations and its small size, our study emphasizes the importance of rapid return of results in critically ill children in this region where the genetic disease burden is expectedly high due to consanguineous marriages [3, 4]. In fact, two of the five patients (40%) had significant ROH (6.5% and 12%), one of whom (patient #2) had a homozygous pathogenic *POMT1* variant within a 13Mb ROH on chromosome 9. Although no clear diagnostic finding was identified in the other patient (patient #4), several genes with homozygous variation within the identified extended ROH can now be candidates for novel gene-disease association and discovery, highlighting the importance of extending such genetic studies to diverse and historically underrepresented patient populations like in the Middle East. The increased accessibility and utilization of standard and rapid WGS will hopefully improve representation in genetic databases over time.

## Supplementary Information

**Additional file 1: Fig. S1.** Schematic representation of Illumina ‘General’ variant filtering and prioritizing strategy. MyKB, Illumina knowledge base database. P, Pathogenic; LP, Likely pathogenic. **Fig. S2.** Schematic representation of ‘Emedgene’ variant filtering and prioritizing strategy. Artificial Intelligent based prioritization of candidate variant was performed using EmedGene, which is available in Illumina TSS. For instance, in one case, a pathogenic homozygous stop gained variant in *POMT1* is associated with muscular dystrophy which is related to patient’s phenotype (patient #2) identified by Emedgene programme. **Fig. S3.** Mosaic tetrasomy 12p13.33p11.1 identified in patient #3. *To*p, Single Nucleotide Polymorphism (SNP) chromosomal microarrays data from paitent #3 showing copy number state, weighted Log2 ratio (using copy number probes), allele difference (using SNP probes), smooth signal, and B-allele frequency across chromosome 12. *Bottom*, SNPs from whole genome sequencing data across chromosome 12 for patient #3, her mother, and father. The 12p amplification is clearly evident in patient #3.

## Data Availability

All data used and generated is available within the main manuscript and supporting files. Patients were not consented to share the raw WGS data files beyond the research and clinical teams.
