# Supplementary material for: Rapid whole genome sequencing of critically ill pediatric patients from genetically underrepresented populations
Source: Genome Med. 2022 May 24;14:56. doi: 10.1186/s13073-022-01061-7 (PMC9128109; doi:10.1186/s13073-022-01061-7)
Supplement: Supplementary file 1 — Additional file 1: Fig. S1. Schematic representation of Illumina ‘General’ variant filtering and prioritizing strategy. MyKB, Illumina knowledge base database. P, Pathogenic; LP, Likely pathogenic. Fig. S2. Schematic representation of ‘Emedgene’ variant filtering and prioritizing strategy. Artificial Intelligent based prioritization of candidate variant was performed using EmedGene, which is available in Illumina TSS. For instance, in one case, a pathogenic homozygous stop gained variant in POMT1 is associated with muscular dystrophy which is related to patient’s phenotype (patient #2) identified by Emedgene programme. Fig. S3. Mosaic tetrasomy 12p13.33p11.1 identified in patient #3. Top, Single Nucleotide Polymorphism (SNP) chromosomal microarrays data from paitent #3 showing copy number state, weighted Log2 ratio (using copy number probes), allele difference (using SNP probes), smooth signal, and B-allele frequency across chromosome 12. Bottom, SNPs from whole genome sequencing data across chromosome 12 for patient #3, her mother, and father. The 12p amplification is clearly evident in patient #3. [file 13073_2022_1061_MOESM1_ESM.docx]

Methods

**Patient recruitment**

Patients with complex medical findings that could not otherwise be explained by non-genetic factors were identified by the neonatal or pediatric intensive care unit (ICU) teams as candidates for enrollment in this study. The ICU head contacted the genetic counseling department to confirm eligibility. Selected candidates in the ICU setting were either not stable with complex medical findings that needed a rapid diagnosis to aid in treatment or were admitted for an extended period with no improvement and all previous testing was comprehensive yet inconclusive. Patients with findings suggestive of known genetic conditions (such as DiGeorge syndrome or Trisomy 21) were not selected for enrolment and were more effectively diagnosed with targeted testing for these conditions. Once patients were identified by the care team for enrollment, the genetic counselor consented and enrolled the family. An informed consent was obtained according to a protocol approved by the Dubai Healthcare Authority Research Ethics Committee (AJCH-68).

**Sample collection**

1-3ml of blood was collected from each patient and their parents in an EDTA tube. All samples were then immediately processed for automated DNA extraction using the QIAsymphony system (QIAGEN, Germany).

**Library preparation and Sequencing**

Sequencing libraries were prepared using the Illumina DNA PCR-Free Library Prep, Tagmentation (Illumina, USA). Around 300-2000ng of genomic DNA was used from each sample for an initial DNA tagmentation using Bead-Linked Transposomes (BLT-PF) which fragment and tag the DNA with adapter sequences. Following ligation of Index 1 (i7) and Index 2 (i5), the final libraries were then purified by a double-sided bead purification procedure and quantified to a final loading concentation of 1.1nM by a KAPA qPCRLibrary Quantification Kit (Roche, Switzerland).

Libraries from each family (Trios) were pooled and then sequenced using paired end mode (2X150bp) on an S1 flow cell and the NovaSeq system (Illumina, USA) to generate, on average, 182 GB of data and an autosomal coverage of >30X (**Table S3**).

**Read mapping and variant calling**

The NovaSeq run was demultiplexed using Illumina BCL convert application version 2.0.0^1^. The resultant demultiplexed fastq files corresponding to each sample (Proband, Mother and Father) were then transferred to Illumina TruSight Software Suite Version 2.0.0 (TSS)^2^ for secondary and tertiary analysis. The TSS uses DRAGEN^TM^ Bio-IT platform (v3.7.5) which consists of optimized algorithms for alignment and variant calling. The demultiplexed reads were mapped to the GRCh37 human genome. The mapped reads were sorted, duplicates were removed, and variants were called using DRAGEN^TM^ Joint Genotyping Pipeline. In addition to SNVs and INDELs, DRAGEN^TM^ pipeline called structural variants (SVs), mitochondrial variants, repeat expansions, runs of homozygosity (ROH), and *SMN1/SMN2* variants.

**Variant annotation and prioritization**

TSS provides several variant annotations to aid in filtering down to candidate variants. The identified variants were annotated from publicly available databases, such as refseq^3^, OMIM^4^, ClinVar^5^ and gnomAD^6^ using TSS. These annotations provide insights related to a variant’s prevalence in the population, potential involvement in human disease and phenotypes, and predicted impact to protein function. After annotation, variants were filtered and prioritized using Illumina ‘General’ (**Fig. S1**) and ‘Emedgene’ (**Fig. S2**) filtering strategies (**Fig. S1** and **S2**). The latter takes into consideration, among other features, protein effect, inheritance model and, most importantly, patient phenotype to rank variants.

We evaluated small (SNVs, Indels) and large variants (SVs, CNVs, ROH and STR) retained through the ‘General’ filtering strategy (**Fig. S1**). Rare (<0.01 MAF), high quality (PASS) variants consistent with recessive or *de novo* inheritance were retained in this filter. For small variants, we retained those which were coding in genes with association to disease (OMIM), and which do not have a benign or likely benign assertion in ClinVar while variants with a pathogenic or likely pathogenic assertion in ClinVar and Illumina MyKB database were prioritized in this bin. In addition, we retained noncoding variants with a splice AI score > 0.5. For large variants, were retained rare SVs defined as indels >50bp, translocations, and CNVs >10kb overlapping transcripts with consequences such as transcript amplification, transcript ablation and feature truncation (**Fig. S1**).

The ‘EmedGene’ filter utilizes an artificial intelligence (AI) tool to prioritize most-likely candidate variants. It generates a knowledge-graph showing supporting evidence for the variant prioritization using disease–gene relationships, generated by evaluating phenotypes, inheritance modes, splicing predictions, conservation, etc., and by the application of natural language processing (NLP) to various data sources (**Fig. S2**). Patient phenotype is central to ranking variants in this filter.

Refer to **Table S4** for total number of variants in each sample, and the final filtered/prioritized variants through the General’ and ‘Emedgene’ filtering strategies.

**Variant curation and molecular review**

Prioritized variants were manually curated by a genomic scientist, a genetic counselor and an American Board of Medical Genetics and Genomics board certified molecular geneticist.

Clinically relevant variants were immediately reviewed with the care team to reach a consensus. Variants confirmed to be pathogenic or likely pathogenic explaining the patient’s phenotype were clinically confirmed on site in the CAP-accredited Clinical Genomics facility within Al Jalila Children’s Specialty Hospital.

**Return of Results**

Results were communicated verbally to the care team by the genetics laboratory director or the genetic counselor. The genetic counselor reviewed the results with all of the families and in the case of a positive result a multidisciplinary team meeting was arranged with the family to review the results and outline treatment plans. For the cases that were positive, consent was obtained for confirmation testing on a clinical basis. The results of confirmatory clinical test were shared with the medical team and directly with the family.

**Supplementary Figures**


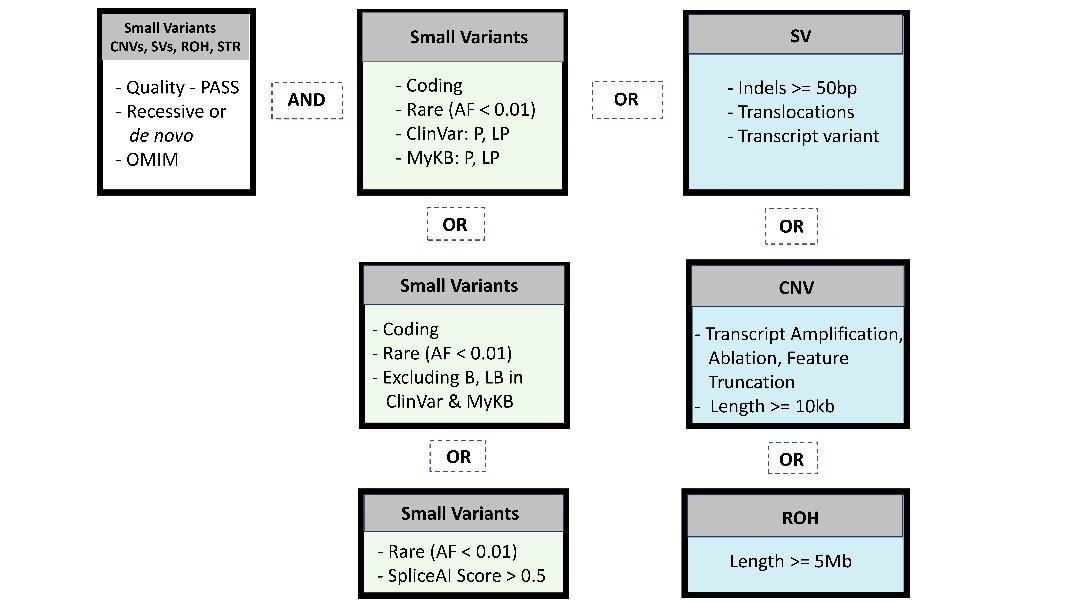
**Fig. S1**


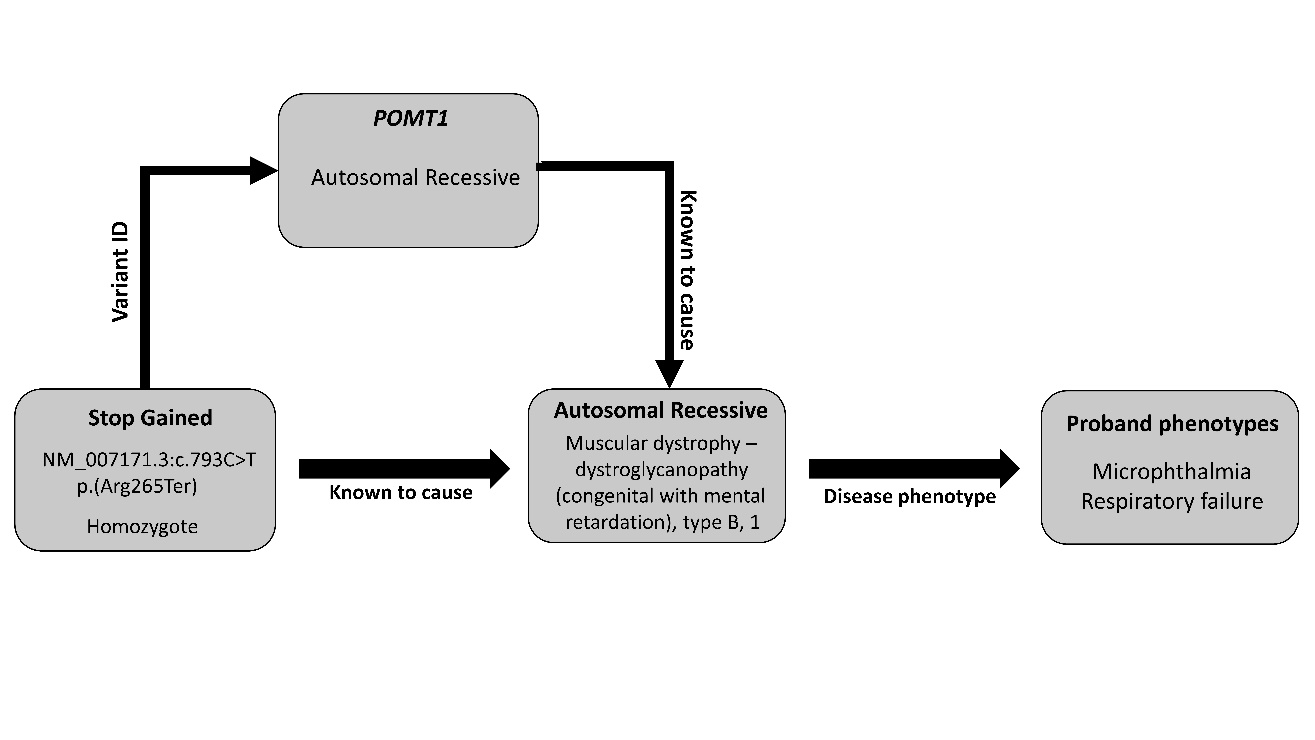
**Fig. S2**


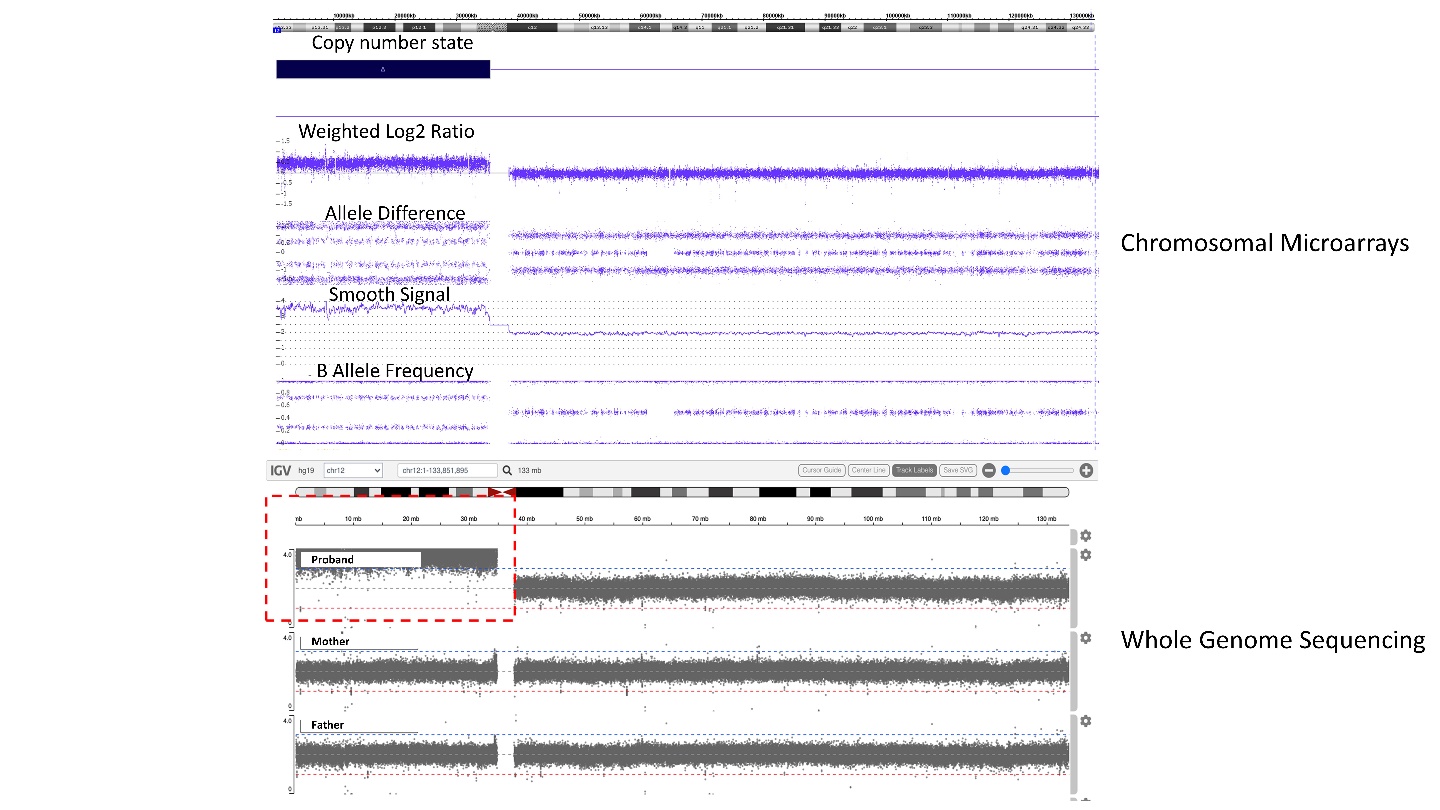
**Fig. S3**

**References:**

1. <https://support.illumina.com/sequencing/sequencing_software/bcl2fastq-conversion-software.html>
2. <https://emea.illumina.com/products/by-type/informatics-products/trusight-software-suite.html>
3. Pruitt, KD, Brown, GR, Hiatt, SM, Thibaud-Nissen, F, Astashyn, A, Ermolaeva, O, Farrell, CM, Hart, J, Landrum, MJ, McGarvey, KM, et al. RefSeq: An Update on Mammalian Reference Sequences. *Nucleic Acids Res.* 2014;(*42)*:756–763.
4. Online Mendelian Inheritance in Man, OMIM®. McKusick-Nathans Institute of Genetic Medicine, Johns Hopkins University (Baltimore, MD, USA). Available online: <https://omim.org/>
5. Landrum, MJ, Lee, JM, Riley, GR, Jang, W, Rubinstein, S, Church, DM, Maglott, DR. ClinVar: Public Archive of Relationships among Sequence Variation and Human Phenotype. *Nucleic Acids Res.* 2014;(*42*)980–985.
6. Karczewski, KJ, Francioli, LC, Tiao, G, Cummings, BB, Alfoldi, J, Wang, Q, Collins, RL, Laricchia, KM, Ganna, A, Birnbaum, DP, et al. The Mutational Constraint Spectrum Quantified from Variation in 141,456 Humans. *Nature* 2020; (*581):*434–443.

| **Table S1.** Patient demographics and clinical presentation |
| --- |

| **Patient** | **Age** | **Gender** | **Nationality** | **Clinical Presentation** | **Consanguinity (Yes/No)** |
| --- | --- | --- | --- | --- | --- |
| #1 | 30 days | Male | Kenyan | Acidosis, Failure to thrive, Development delay, Proteinuria, Kyphosis | No |
| #2 | 2 days | Female | Jordanian | Respiratory failure, Microphthalmia of both eyes, Anorectal malformation, Congenital dilated renal pelvis | Yes - Parents are second cousins |
| #3 | 1 day | Female | Pakistani | Dysmorphic features, Hypoplastic right heart, History of imperforate anus, Acute respiratory failure | No |
| #4 | 18 days | Female | Emirati | Aorta coarctation, Acute hypoxemic respiratory failure, Hypoglycemia, Hypokalemia, Hypothyroidism | Yes - Parents are first cousins |
| #5 | 3 months | Female | Filipino | Hemophagocytic lymphohistiocytosis, Jaundice, Hepatomegaly, Thrombocytopenia, Splenomegaly | No |

|  | | | | |  |  |  |  |  |  |  |  |
| --- | --- | --- | --- | --- | --- | --- | --- | --- | --- | --- | --- | --- |
| **Table S2.** Step-wise run time (hours) | | | | | | |  |  |  |  |  |  |
|  | | | | | | |  |  |  |  |  |  |
|  | **Wet bench (hours)** | | | | | **Bioinformatics and interpretation (hours)** | | | | | | **Total Time** |
| **Patient** | **DNA Extraction** | **Library preparation** | **Sequencing** | **Total Wet bench time** | | **Demultiplexing** | | **Alignment and variant calling** | **Annotation** | **Variant Interpretation** | **Total Dry bench time** |  |
| #1 | 1 | 4 | 23.48 | 28.48 | | 1.59 | | 3.2 | 1.08 | 5 | 10.87 | 39.35 |
| #2 | 1 | 4 | 23.48 | 28.48 | | 1.1 | | 3.23 | 0.98 | 2 | 7.31 | 35.79 |
| #3 | 1 | 4 | 23.23 | 28.23 | | 1.29 | | 3.7 | 0.92 | 2 | 7.91 | 36.14 |
| #4 | 1 | 4 | 24.47 | 29.47 | | 1.2 | | 3.27 | 1.1 | 4 | 9.57 | 39.04 |
| #5 | 1 | 4 | 24.47 | 29.47 | | 1.23 | | 3.18 | 1.13 | 1.5 | 7.04 | 36.51 |
| **Average (hours)** | **1** | **4** | **23.826** | **28.826** | | **1.282** | | **3.316** | **1.042** | **2.9** | **8.54** | **37.366** |

| **Table S3.** Whole genome sequencing data quality metrics | | | | |  |  |  |  |
| --- | --- | --- | --- | --- | --- | --- | --- | --- |
|  |  | **Data (GB)** | **% Passed Filter** | **% Q30** | | **Total # of reads** | **% Reads aligned** | **Average autosomal coverage over genome** |
| **Family 1** | **Proband** | 202.11 | 78.18 | 89.66 | | 1,338,453,702 | 94.13 | 52.2 |
|  | **Mother** | 169.11 |  | 89.44 | | 1,119,953,874 | 94.59 | 43.5 |
|  | **Father** | 198.81 |  | 89.4 | | 1,316,623,832 | 94.46 | 53.18 |
| **Family 2** | **Proband** | 216.62 | 70.74 | 90.01 | | 1,434,584,518 | 94.51 | 53.87 |
|  | **Mother** | 144.06 |  | 90.42 | | 954,035,904 | 94.8 | 35.27 |
|  | **Father** | 148.25 |  | 90.43 | | 981,788,702 | 94.22 | 37.47 |
| **Family 3** | **Proband** | 186.46 | 78.72 | 89.73 | | 1,234,839,164 | 94.58 | 47.94 |
|  | **Mother** | 204.84 |  | 91.25 | | 1,356,542,336 | 94.91 | 52.59 |
|  | **Father** | 183.96 |  | 90.08 | | 1,218,308,374 | 94.46 | 47.96 |
| **Family 4** | **Proband** | 187.58 | 71.24 | 89.76 | | 1,242,221,654 | 94.77 | 41.35 |
|  | **Mother** | 161.41 |  | 89.22 | | 1,068,953,726 | 94.69 | 61.14 |
|  | **Father** | 175.65 |  | 89.8 | | 1,163,248,444 | 94.46 | 41.18 |
| **Family 5** | **Proband** | 159.41 | 76.49 | 89.72 | | 1,055,702,740 | 94.56 | 47.68 |
|  | **Mother** | 232.38 |  | 89.5 | | 1,538,910,000 | 94.79 | 42.83 |
|  | **Father** | 157.64 |  | 89.56 | | 1,043,979,292 | 93.83 | 46.85 |
| **Average** |  | **181.89** | **75.07** | **89.87** | | **1,204,543,084.13** | **94.52** | **47.00** |

| **Table S4.** Variant calls   \| **Patient** \| **Consanguinity (Yes/No)** \| **Total # variants** \| **Prioritized SNVs/INDELs*** \| **Prioritized SVs**** \| **ROH** \| \| --- \| --- \| --- \| --- \| --- \| --- \| \| #1 \| No \| 5,605,795 \| 10 \| 12 \| NS \| \| #2 \| Yes - Parents are first cousins \| 5,036,258 \| 10 \| 15 \| 6.56% \| \| #3 \| No \| 5,076,374 \| 10 \| 26 \| NS \| \| #4 \| Yes - Parents are first cousins \| 4,824,863 \| 10 \| 22 \| 12% \| \| #5 \| No \| 4,971,492 \| 10 \| 8 \| NS \|   *SNVs/INDELs prioritized in the Emedgene bin. **prioritized in the General bin (See Supplemental Methods). SNVs, single nucleotide variants; INDELs, small insertions and deletions; SVs, structural variants including copy number variants and short tandem repeats; ROH, regions of homozygozity |
| --- | --- | --- | --- | --- | --- | --- | --- | --- | --- | --- | --- | --- | --- | --- | --- | --- | --- | --- | --- | --- | --- | --- | --- | --- | --- | --- | --- | --- | --- | --- | --- | --- | --- | --- | --- | --- |

| **Table S5.** Diagnostic Findings | | | | |  |  |  |  |  |  |  |
| --- | --- | --- | --- | --- | --- | --- | --- | --- | --- | --- | --- |
| **Patient** | **Age** | **Gender** | **Nationality** | **Clinical Presentation** | | **Gene** | **HGVS/ISCN Nomenclature** | **Zygosity** | **ACMG Classification (Codes)** | **Confirmatory clinical Testing** | **Diagnosis** |
| #2 | 2 days | Female | Jordanian | Respiratory failure, Microphthalmia of both eyes, Anorectal malformation, Congenital dilated renal pelvis | | *POMT1* | NM_007171.3:c.793C>T;p.(Arg265Ter) | Homozygous | Pathogenic (PVS1, PP4, PM3, PM2) | Clinical Exome Sequencing | Muscular dystrophy-dystroglycanopathy |
| #3 | 1 day | Female | Pakistani | Dysmorphic features, Hypoplastic right heart, History of imperforate anus, Acute respiratory failure | | 12p13.33p11.1 | arr[GRCh37] 12p13.33p11.1(173787_34835837)x3-4 | Mosaic tetrasomy | NA | Chromosomal Microarrays | Pallister-Killian syndrome (PKS) |
| #5 | 3 months | Female | Filipino | Hemophagocytic lymphohistiocytosis, Jaundice, Hepatomegaly, Thrombocytopenia, Splenomegaly | | *LIPA* | NM_000235.3:c.521C>T;p.(Ser174Phe)  NM_000235.3:c.193C>T;p.(Arg65Ter) | Compound Heterozygous | Likely Pathogenic (PM1, PM2, PP4, PP3)  Pathogenic (PVS1, PM3, PM2) | Sanger Sequencing | Lysosomal Acid Lipase Deficiency (Wolman Disease) |
